# Effectiveness and Moderators of Wise Interventions in Reducing Depressive and Anxiety Symptoms Among Youth: A Systematic Review and Meta-analysis of Randomised Controlled Trials

**DOI:** 10.1007/s10578-025-01832-4

**Published:** 2025-04-07

**Authors:** Melisa Parlak, Gonzalo Salazar de Pablo, Patrick Nyikavaranda, Matthew Easterbrook, Daniel Michelson

**Affiliations:** 1 School of Psychology, University of Sussex, Brighton, UK; 2 Department of Child and Adolescent Psychiatry, Institute of Psychiatry, Psychology and Neuroscience, King’s College London, London, UK; 3 NIHR Maudsley Biomedical Research Centre, South London, London, UK; 4 Primary Care and Public Health Department, Brighton and Sussex Medical School, Brighton, UK

**Keywords:** Youth mental health, Wise Interventions, Randomised controlled trials, Systematic review, Meta-analysis

## Abstract

Wise interventions (WIs) use theory-driven approaches to reshape individuals’ interpretations of their experiences. In these pre-registered meta-analyses, we conducted random-effects, fixed-effects, moderation, and subgroup meta-analyses across different time points to evaluate the effects of WIs on depressive or anxiety symptoms. We also conducted quality assessments and evaluated publication bias and heterogeneity. Sixteen RCTs were included, revealing small but significant effects of WIs on depressive symptoms post-intervention (g = 0.22; p = 0.00) and anxiety symptoms at post-intervention (g = 0.20; p = 0.00) and 3-month follow-up (g = 0.09; p = 0.02). The strongest post-intervention effects on depressive symptoms were found for gratitude interventions (g = 0.29; p = 0.04) and online delivery (g = 0.35; p = 0.03). Moderation analyses for other endpoints yielded equivocal results. These findings highlight new opportunities to support youth by reframing their identities as sources of strength and fostering gratitude.

**Supplementary Information:**

The online version contains supplementary material available at https://doi.org/10.1007/s10578-025-01832-4.

## Introduction

Youth mental health problems are common and potentially increasing. In the UK, the proportion of 17–19-year-olds experiencing probable mental disorders rose from 10.1% in 2017 to 17.7% in 2020, reaching 23.3% in 2023 [42, 43]. Treatment gaps for common mental health disorders (CMD) among youth are about 88% in low- and middle-income countries (LMICs; [16, 82]) and around 40% in high-income countries (HICs). Despite being lower in HICs, this gap remains significant for young people compared to other age groups. Limited or non-existent child and adolescent mental health services in HICs further complicate meeting this demand [76]. Factors contributing to the global treatment gap include stigma, limited mental health literacy and under-resourced healthcare systems [19, 26, 32, 47, 53, 74]. To address this gap, it is essential to implement effective and accessible interventions that prevent the onset, development, and persistence of mental health issues, which can lead to long-term consequences such as academic underachievement and unemployment [23, 41, 84].

Growing attention has focused on Wise Interventions (WIs) as a potentially scalable approach for mental health prevention and early intervention [61]. WIs are brief, theory-driven strategies designed to change how individuals attribute meaning to their everyday experiences, thus fostering adaptive behaviour change [81]. The term “Wise” originates from Goffman [24], who used it to describe individuals who understood and sympathised with the experiences of gay individuals, even if they did not share those stigmatised identities themselves. Steele [66] described “wise schooling,” which addresses the experiences of students facing negative stereotypes in academic settings. From these foundations, Walton and Wilson [81] defined WIs as comprising “psychologically wise” strategies that reshape individuals’ interpretations of themselves, others, and their circumstance. Historically, WIs have focused on educational outcomes, while also showing positive effects on youth relationships and resilience [79, 81, 90]. WIs have increasingly been used to address mental health outcomes, especially among young people [61]. Common ingredients include growth mindset, self-affirmation, and social belonging strategies (see examples in Table 1).

**Table 1:** Common ingredients for WI (from Ref. [79])

Ingredient	Example
Growth mindset	In classrooms, high school students learn that intelligence is flexible and can grow through effort and support, allowing them to enhance their learning rather than view it as a fixed limitation [8, 91]
Social belonging	First-year college students are reassured that initial concerns about belonging and daily challenges are normal during the transition to college, and that, over time, everyone eventually finds a sense of belonging and comfort [80]
Self-affirmation	Students engage in in-class activities where they reflect on the importance of their core values, such as relationships with friends and family, which boosts their self-integrity and helps prevent negative cycles of threats and poor academic performance [63]
Gratitude	Students reflect on and write about their daily gratitude at home, enhancing their self-integrity and fostering a more optimistic perspective [65]

Recent research highlights key differences between WIs and traditional evidence-based treatments (EBTs) for mental health problems. WIs are brief, often involving just one or a few sessions, which has the potential to increase uptake for young people who may be deterred by longer treatments assuming they can access such high-intensity interventions in the first place [84]. Unlike EBTs, which use multi-component approaches like cognitive-behavioural therapy, WIs typically focus on a single theorised active ingredient^1^ [89]. This can simplify delivery by lay providers, while also being conducive to scalable intervention formats that utilise psychologically precise, self-directed activities [39, 58, 61].

WIs target three core psychological needs—belonging, self-integrity, and understanding [81]—and by effectively engaging these needs, they promote adaptive behaviour changes that reinforce positive belief shifts, ultimately enhancing mental health outcomes. These interventions promote adaptive interpretations of personal experiences and reduce rumination and repetitive negative thinking, which are key risk factors for anxiety and depression [52, 72]. Specifically, by fostering a sense of social connectedness to address *the need to belong,* WIs help individuals feel cared for, thereby mitigating the isolation often associated with depression [12]. Additionally, by bolstering *self-integrity*, they support a resilient sense of self-worth, preventing the anxiety that can arise from negative stereotypes. Finally, WIs that cultivate an adaptive *understanding* of stressors—for example, by promoting a growth mindset—enable individuals to reframe setbacks constructively, reducing feelings of helplessness and distress [50]. Collectively, these mechanisms help disrupt maladaptive threat-based thinking and support improved mental health outcomes.

We aimed to carry out the first meta-analysis that focused specifically on the effects of WIs on youth anxiety and depression, which are the two most common mental health problems faced by 10–24-year-olds [38]. As noted earlier, WIs have distinct active ingredients, yet their shared aim of shifting individuals’ interpretations of themselves or their situations [81] support their inclusion together in a meta-analysis of WIs on youth depression and anxiety, despite focusing on different psychological needs such as belonging or self-integrity. In alignment with Walton and Wilson’s [81] definition of WIs, we employed stringent inclusion and exclusion criteria rooted in their theoretical framework to ensure conceptual precision. Additionally, the first and only review of different WIs in youth mental health was conducted five years ago (i.e., [61]), and an update is overdue with more rigorous criteria, with a novel aspect of this work being a meta-analysis to evaluate WIs’ effectiveness. This research also builds on previous reviews of WIs’ effectiveness on youth mental health [10, 35, 57], which reported inconsistent findings or focused on only one type of WI at a time. Moreover, by evaluating the impacts of WIs at multiple time points—post-intervention and at 3- and 6-month follow-ups—this review addresses the gaps identified by Schleider et al. [61] and Burnette et al. [10], particularly the lack of detailed data and insufficient timing of assessments, which hinder the ability to analyse effects over time.

Additionally, understanding the factors that shape the effectiveness of WIs is key to enhancing their implementation and improving outcomes, as highlighted in social psychology research [13]. Specifically, we focused on study-specific characteristics, as other meta-analyses have shown that these factors moderate outcomes related to mental health and academic performance (e.g., [58, 88]). The research questions of the current review are: (1) What are the effects of WIs on depressive and anxiety symptoms for young people aged 10–24 years? (2) To what extent are these effects moderated by intervention characteristics, including type, setting, frequency, method of delivery?

## Method

### Registration

The protocol for this review was pre-registered on PROSPERO (reference: CRD42023398601^2^; date of registration: 22/02/2023). We followed the PRISMA [40] and MOOSE (Meta Analyses of Observational Studies in Epidemiology; [69]) checklists and reporting guidelines (Online Resource 1).

### Search Strategy

We conducted electronic searches in six databases (ProQuest, PsycINFO, Medline, Eric, Scopus, PubMed), as well as conducting manual forward/backward citation searching of relevant literature to eligible studies published up to 22/08/2023, with no limit placed on the earliest possible date of publication. Boolean operators were used with four categories of search terms: youth populations, WIs, depressive and anxiety symptoms, and randomised controlled trials. When full texts were unavailable, the first author contacted the study authors to request access to the complete documents. The tabulated search terms are provided in the Online Resource 2.

### Inclusion and Exclusion Criteria

Study inclusion criteria were: (1) participants aged between 10–24 years; (2) one of the groups assigned to a wise intervention; (3) the presence of a control group; (4) assessment of depressive or anxiety symptoms at baseline and one or more endpoints (regardless of whether anxiety/depression was the primary outcome); (5) randomised controlled trial (RCT) design; and (6) published in English and peer-reviewed. Additionally, we excluded studies that combined multiple WI components (e.g., growth mindset and gratitude).

To qualify as a Wise intervention, we applied three criteria [81]. First, the intervention must be built around a single active ingredient, i.e., incorporating a single conceptually well-defined component that links to specific hypothesised mechanisms of action [87]. For each study, we identified the specific theory used to assess whether the intervention was constructed around a singular, well-defined active ingredient that directly corresponded to the hypothesised mechanisms of action. This evaluation involved a systematic examination of the theoretical framework detailed in each paper, with any discrepancies resolved through consensus between the first author and senior supervisors. Second, the targeted mechanisms should involve shifting participants’ perceptions of themselves, their social environment, or their beliefs, emphasising understanding, belonging, and self-integrity [81]. This would exclude interventions focused on behavioural skills or cognitive processes, such as cognitive bias modification and cognitive dissonance, as they do not foster deeper transformations in self-concept or social understanding. Finally, given we want to include scalable WIs that also have potential for dissemination, we restricted interventions to a maximum duration of 240 min, equivalent to four 60-min sessions [81].

### Selection

After removing the duplicates, initial eligibility screening was conducted by the first author, based on assessment of abstracts and titles using EndNote bibliographic software [18]. The full texts of the remaining articles were assessed for eligibility by the first author. Independent screening was conducted independently by a second reviewer (PN) for eight full-text papers selected at random. Cohen’s kappa for agreement was κ = 0.788, reflecting moderate inter-rater agreement [37]. In instances of disagreement regarding study inclusion or exclusion, both reviewers articulated their rationale based on established criteria. The senior co-author (M.J.E.) then evaluated these arguments in the context of the study’s objectives and consulted the first author to resolve ambiguities before rendering a final decision.

### Data Extraction

The characteristics of included studies were coded by the first author using a structured proforma, covering the following domains: first author and year of publication, country, sample size, age in years (mean ± s.d.), sex (% female), ethnicity (% White), core ingredient, setting, duration, method of delivery, control condition, reported depressive and anxiety symptoms along with their measures, and endpoints.

### Quality Assessment

We assessed study quality using the Cochrane Collaboration’s RoB 2 tool [67], evaluating five domains: randomisation process, deviations from intended interventions, missing outcome data, outcome measurement, and selection of reported results. Each domain was rated as "low risk of bias," "some concerns," or "high risk of bias," leading to an overall bias risk classification. Studies were categorised as "low risk" if all domains showed low bias, "some concerns" if at least one domain raised concerns without reaching high bias risk, and "high risk" if at least one domain indicated high risk or multiple concerns undermined confidence in the results. Bias scoring was conducted independently by MP and PN for all papers, with Cohen’s kappa indicating moderate inter-rater agreement (κ = 0.696). Disagreements were resolved through discussions.

### Data Synthesis and Analysis

We conducted a narrative synthesis structured around WI characteristics. To examine the effects of WIs (relative to control conditions) on depressive and anxiety symptoms, meta-analyses were conducted using Hedges’ g across three endpoints: post-intervention, 3- and 6-month follow-ups for each symptom cluster separately. Effect sizes were classified as small (d = 0.20), medium (d = 0.50) or large (d ≥ 0.80), following the conventions described by Sullivan and Feinn [70].

To address variability across studies, we conducted heterogeneity analyses to determine the appropriate model for each endpoint and outcome. We examined heterogeneity among study estimates using Q statistics, with the I^2^ index assessing the proportion of variability in effect size estimates, classifying the heterogeneity as into low (I^2^ = 25%), medium (I^2^ = 50%), and high (I^2^ = 75%) [27],and significance was set at p < 0.05. For endpoints with low heterogeneity (I^2^ ≤ 25%), we employed a fixed-effects model. In cases of medium and high heterogeneity (I^2^ > 50%), we used a random-effects model to account for study variability. Additionally, for the random-effects analyses, prediction intervals were calculated to provide a range for the expected true effect sizes. When the number of studies exceeded seven, we assessed publication bias through funnel plots and Egger’s test [17], applying the "trim and fill" method to adjust for potential missing studies related to publication bias, such as small sample bias [55].

Given the limited sample size across various endpoints for depressive and anxiety symptoms, it was not feasible to analyse multiple moderators within a single model. Therefore, we conducted a series of models that varied the reference group to estimate all potential contrast effects across the following domains. For example, for depressive symptoms at the post-intervention time point, we examined: (1) core ingredient (“self-affirmation,” “social belonging,” “gratitude”); (2) setting (“online,” “school,” “laboratory,” “home”); (3) frequency (“single” vs. “multiple”); and (4) method of delivery (“guided” vs. “self-administered”). We carried out moderator analysis for a particular symptom cluster/endpoint when at least eight studies were available within a given domain [2]. Additionally, we ran separate models for each intervention characteristic at the respective time points, and compared their effect to further validate our findings. Comprehensive Meta Analysis (CMA) version 4 [9] was utilised for the analyses.

## Results

**Fig. 1:**
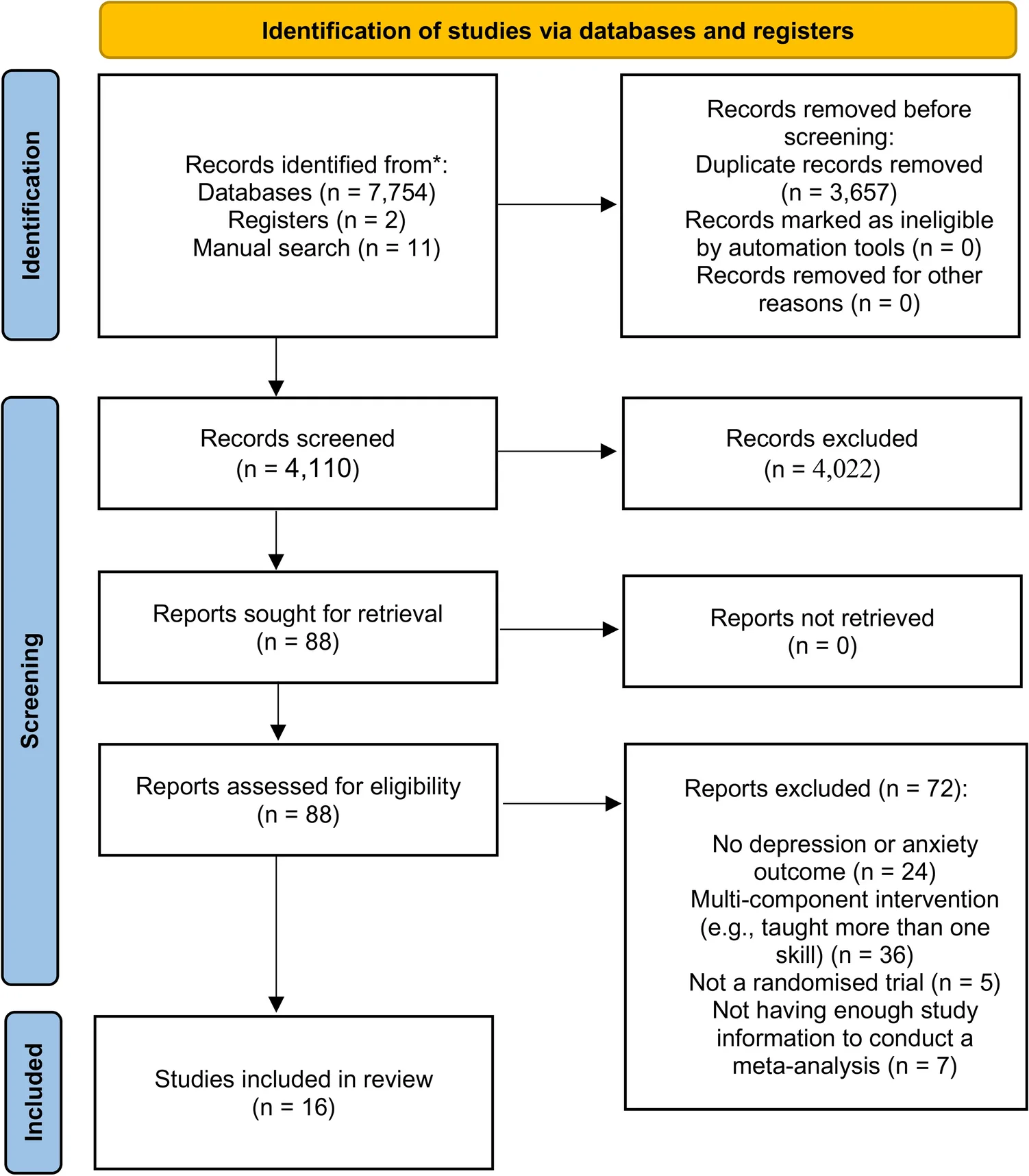
PRISMA 2020 Flow Diagram [49]

The initial search yielded 7,767 results, of which 3,657 were duplicates and subsequently removed. The remaining 4,110 citations were then screened for eligibility by the first author, following which 88 full-text reports were retrieved. From these, 72 were excluded (see PRISMA diagram in Fig. 1). The assessments and exclusions were made by the first author in consultation with a senior co-author (MJE). The final database for the meta-analysis comprised 16 studies. Detailed study characteristics can be found in Online Resource 3.

### Study Characteristics

The total sample included 4,471 participants, with 2,301 (51.46%) in the intervention groups and 2,170 (48.54%) in the control groups. Study characteristics are shown in Online Resource 3. The studies included samples from nine countries, with half (k = 8) conducted in the USA and single studies from Australia, Germany, India, Kenya, New Zealand, Poland, Spain, and the UK. Participants’ mean ages ranged from 12.2 years (*SD* = *1.8*) to 23.68 (*SD* = *3.11*). In 14 of the 16 studies, females were the majority, with two exclusively targeting female participants [60, 86]. Across all studies, the percentage of female participants ranged from 42.5% to 100%. White participants were the majority in 10 of the 12 studies reporting ethnicity, with representation varying from 0% (in a study exclusively targeting Indian youth; [65]) to 92.5% [36].

The included WIs incorporated four core components: growth mindset (k = 6), gratitude (k = 6), self-affirmation (k = 3), and social belonging (k = 1). Seven studies were conducted in person—three in school settings and four in laboratory settings—while nine studies took place at participants’ homes (k = 4) or online (k = 5). The majority of studies were self-administered (k = 14), with four using computer-based formats and 10 utilising paper materials. The remaining two studies were guided: one led by psychologists, involving three intervention groups divided by school year, and the other facilitated by recent high school graduates.

Nine studies involved single-session WIs lasting 15 to 60 min, while the remaining seven included multiple sessions, mostly gratitude-based (k = 6), where participants reflected on gratitude over one to eight weeks. Additionally, one self-affirmation intervention had 20-min sessions over three days. Half the studies (k = 8) used attention control groups, replicating intervention content and engagement. Five used active controls, such as supportive therapy [68] or a computer-based single-session intervention (SSI) promoting healthy sexual behaviour (HEART [85],). Three studies used passive controls, such as standard care without treatment.

Depressive symptoms were assessed in 15 of the 16 RCTs, with 10 showing significant reductions, primarily in gratitude-based WIs (k = 5). Reliable, validated self-report measures assessed depressive symptoms, including the General Health Questionnaire-28 (GHQ-28; [25]) and the Center for Epidemiological Studies Depression Scale for Children (CES-DC; [83]). Anxiety symptoms were evaluated in 11 studies, with six reporting significant reductions, mostly from gratitude interventions (k = 3). For anxiety symptoms, high-validity measures included, but were not limited to, the Generalised Anxiety Disorder-7 (GAD-7; [64]) and the Screen for Child Anxiety and Related Disorders—Child version (SCARED-C; [7]).

### Quality Assessment and Publication Bias

**Fig. 2:**
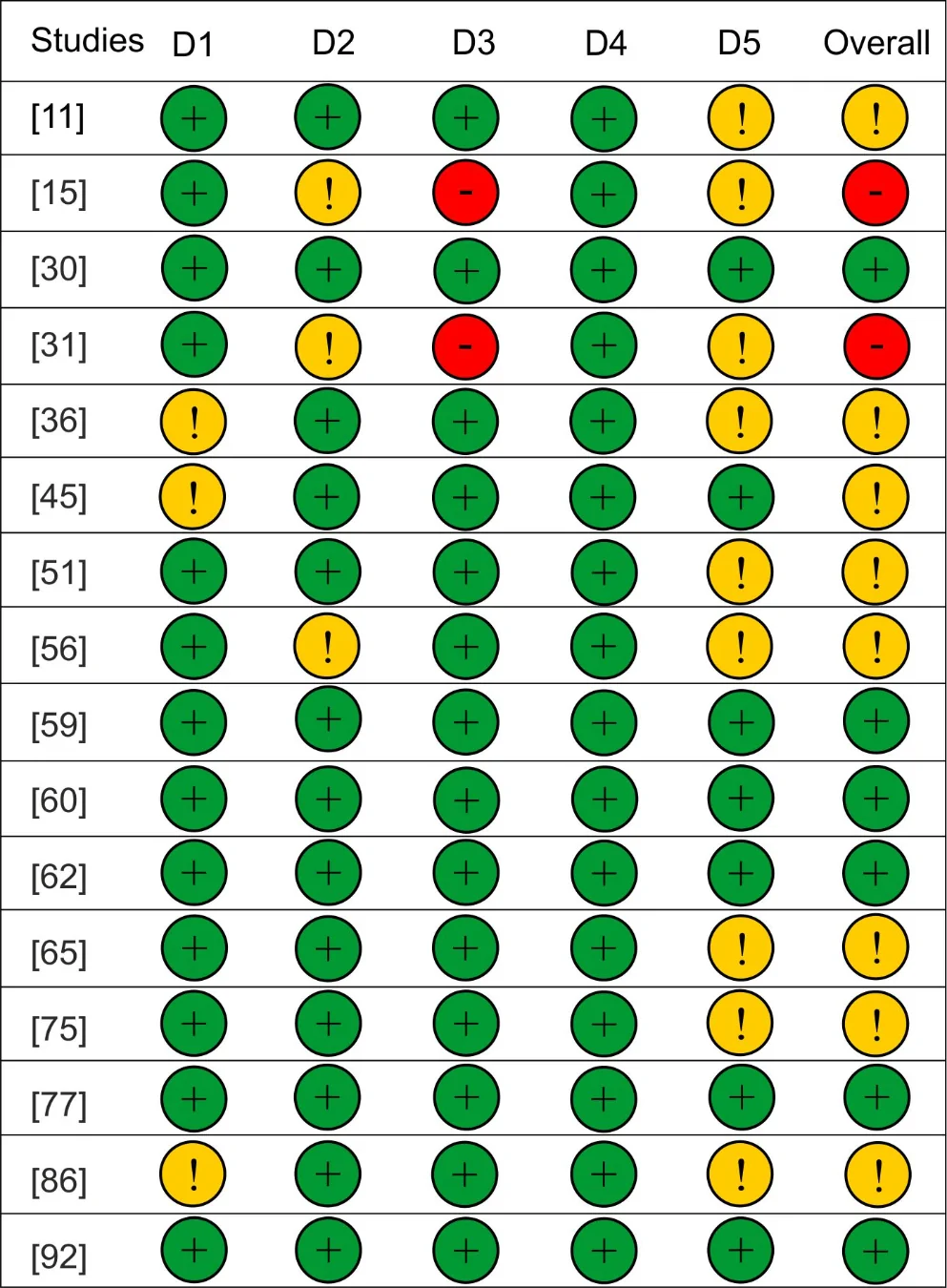
Assessment of Risk of Bias in Included Studies. Risk of bias scoring using Cochrane Collaboration’s RoB 2 tool. Green and red colours correspond to low and high risk of bias, respectively. Yellow represents some concerns. D1 Randomisation process, D2 Deviations from the intended interventions, D3 Missing outcome data, D4 Measurement of the outcome, D5 Selection of the reported result

Six studies (37.5%) were scored as having low risk of bias, eight (50%) as having “some concerns,” and two (12.5%) as having high risk of bias (see Fig. 2). The most commonly noted risks related to insufficient or absent reporting on the extent of missing outcome data. Additionally, a few studies failed to report the randomisation method or the type of tool used, providing only that the study followed an RCT design. There were also concerns about the selection of reported results, specifically regarding whether analyses were conducted according to pre-specified analysis plans. Most studies did not report their pre-specified analysis plans or include trial registration protocol numbers.

### Publication Bias

Funnel plots revealed asymmetry in the data regarding depressive symptoms at post-intervention. At the 3-month follow-up, there was no asymmetry in depressive symptoms. However, the trim and fill method adjusted two values to the left of the mean, resulting in a change in effect size from 0.08 to 0.04, with a 95% CI of [− 0.12, 0.19]. For funnel plots, please see Online Resource 4.

### Effectiveness of WIs

As shown in Table 2, WIs demonstrated small, significant effects on depressive and anxiety symptoms post-intervention, as well as having a very small, significant effects on anxiety symptoms at the 3-month follow-up. WIs did not demonstrate significant effects on either symptom cluster at other endpoints (all p > 0.05). The forest plots illustrating WIs’ effectiveness can be found in Online Resource 5.

**Table 2:** Effectiveness of WIs on depressive and anxiety symptoms

	K	Hedges’ g	95% CIs	p-value	Prediction intervals
Post-intervention depressive symptoms	8	0.22	[0.09, 0.34]	0.001*	
3-month depressive symptoms	8	0.08	[− 0.08, 0.24]	0.34	[− 0.38, 0.54]
6-month depressive symptoms	6	0.10	[− 0.01, 0.21]	0.07	
Post-intervention anxiety symptoms	6	0.20	[0.06, 0.33]	0.001*	
3-month anxiety symptoms	6	0.09	[0.01, 0.18]	0.02*	
6-month anxiety symptoms	2	0.07	[− 0.14, 0.27]	0.52	

*p < 0.05

### Heterogeneity, Moderator and Sub-Analyses

Low heterogeneity was observed for all outcomes/endpoints except for depressive symptoms at 3-month follow-up, which showed medium heterogeneity (I^2^ = 58.198, p = 0.01), requiring a random-effects model. For other outcomes/endpoints, fixed-effects models were applied: depressive symptoms at post-intervention (I^2^ = 14.28, p = 0.32) and 6-month follow-up (I^2^ = 16.01, p = 0.31), as well as anxiety symptoms at post-intervention (I^2^ = 0.00, p = 0.67), 3-month (I^2^ = 9.51, p = 0.36), and 6-month follow-ups (I^2^ = 0.00, p = 0.44).

We conducted moderator analyses to explore the effectiveness of WIs based on their varying characteristics. Significant moderator effects are detailed in Table 3. For each characteristic, such as core ingredient, we conducted a series of models to vary the reference group in order to estimate all potential contrast effects. When examining the effects of different WI ingredients for post-intervention depressive symptoms, our findings revealed that gratitude interventions were significantly more effective than self-affirmation interventions. No other effects reached statistical significance. In terms of setting, the analysis revealed a significant moderation effect: online interventions proved to be more effective than those conducted in school settings. WIs, although this effect was marginal. The analyses revealed non-significant moderating effects for delivery methods and no significant moderating effects for any WI characteristics at the three-month follow-up (all p > 0.05).

**Table 3:** Significant moderator results

WI characteristics	Coefficient	Standard error	95% Lower confidence intervals (CIs)	95% Upper confidence intervals (CIs)	2-sided p value
WI ingredient					
Self-affirmation as the intercept	0.09	0.08	− 0.07	0.26	0.28
Gratitude	0.29	0.14	0.02	0.56	0.04*
Social belonging	0.29	0.23	− 0.16	0.74	0.21
WI setting					
School as the intercept	0.08	0.10	− 0.10	0.27	0.38
Online	0.35	0.16	0.03	0.67	0.03*
Laboratory	0.12	0.19	− 0.26	0.50	0.55
Home	0.17	0.18	− 0.18	0.53	0.33
Duration					
Single-session as the intercept	0.11	0.08	− 0.05	0.28	0.18
Multiple sessions	0.24	0.13	− 0.01	0.50	0.06

*p < 0.05

In addition to the moderator analyses, we explored how WIs performed through subgroup analyses. For example, we conducted separate analyses for each level of WI characteristics, analysed the data across these levels, and compared their effects at different levels of each specific characteristic.

When comparing across outcomes and endpoints for core ingredients, gratitude interventions appeared to have the most promising effect profile post-intervention, as it was the only ingredient to achieve significant effects on both depressive (k = 4; g = 0.38) and anxiety (k = 3; g = 0.30) symptoms. Finding at 3-months found growth mindset (k = 4; g = 0.09) interventions showed significant effects on anxiety symptoms. Findings at 6-months were mixed, with no clear trends favouring any specific ingredient for both outcomes.

Trends also pointed in the direction of online interventions (on depressive symptoms post-intervention; k = 3; g = 0.43), self-administered WIs (on depressive symptoms; k = 7; g = 0.32 and on anxiety symptoms; k = 5; g = 0.19; post-intervention, and on 3-months anxiety symptoms; k = 6; g = 0.09), and multiple-session WIs (on depressive symptoms; k = 5; g = 0.36 and on anxiety symptoms; k = 4; g = 0.24; post-intervention) as showing particular promise. Notably, single-session WIs for anxiety symptoms post intervention (k = 2; g = 0.17) seemed to continue their effects at 3-months (k = 4; g = 0.09). Detailed results from the subgroup analyses, with respective p-values and 95% CIs are available in Online Resource 6.

## Discussion

To the best of our knowledge, this is the first meta-analysis specifically examining the effects of WIs on depressive and anxiety symptoms among youth. Based on 16 RCTs, WIs demonstrated small but significant effects in reducing both depressive and anxiety symptoms at post-intervention. At the 3-month follow-up, a very small significant effect was observed on anxiety symptoms while no effect was observed on depressive symptoms. There was no evidence for any WI effects on depressive or anxiety symptoms at 6-month follow-up. Moderator analyses found that gratitude interventions and online WIs appeared to have the strongest effects on depressive symptoms post-intervention, with small effect sizes. Taken together, these findings suggest the short-term effectiveness of WIs in alleviating depressive and anxiety symptoms, though they also highlight fluctuating heterogeneity across time points and evidence of publication bias. The limited number of studies in diverse contexts also limits our ability to identify specific characteristics that enhance WI effectiveness. Still, our results do allow cautious though valuable conclusions that imply very brief WIs can be effective at reducing anxiety and depressive symptoms.

In their systematic review of WIs and youth psychopathology, Schleider et al. [61] included 25 RCTs, while we identified 16 eligible studies. This is due to our stricter inclusion criteria. We excluded studies that provided personalised feedback on substance use, such as motivational interviewing, or focused on cognitive processes like cognitive bias modification, which did not aim to change participants’ self-concept or social understanding. This aligns with our definition of WIs, which was based on Walton and Wilson [81]. Schleider et al. [61] concluded that WIs could reduce youth psychopathology, particularly noting growth mindset interventions as "Well-Established" for decreasing youth depression. In contrast, our findings show that gratitude interventions significantly reduced depressive symptoms and were more effective than other WI ingredients, while growth mindset interventions only reduced anxiety symptoms at three months with a very small effect size. Thus, while we acknowledge WIs’ potential for addressing youth mental health, our review provides more nuanced insights through significant moderator analyses.

With that in mind, the results of our moderation analyses warrant further discussion. Notably, a meta-analysis by Andrews et al. [1] found that online WIs are as effective as traditional face-to-face treatments, particularly for individuals facing barriers to in-person therapy, such as stigma or logistical challenges [29, 44, 73]. Online interventions are typically less intimidating and more cost-effective [6, 22, 34], though they tend to have higher dropout rates than face-to-face options [20]. However, this concern may be mitigated for WIs, which are often brief (typically a single session), thereby reducing dropout rates. Therefore, further research should compare the effectiveness of online and face-to-face interventions directly.

Our results indicate that multiple-session WIs may have slightly stronger effects on depressive symptoms post-intervention compared to one-off WIs, consistent with evidence that multiple-session psychotherapies are generally more effective in reducing mental health issues [54]. Although SSIs, which extend beyond single sessions, have effectively reduced anxiety symptoms [58], addressing depression may benefit from a multi-session approach for optimal outcomes. Furthermore, although our review included more studies on self-administered WIs than guided WIs, moderator analyses did not show significant results despite significant effects observed in subgroup analyses. This suggests that guided WIs may demonstrate stronger effects if additional studies were available. This is supported by a meta-analysis of 50 RCTs, which found that guided SSIs had greater impacts on youth psychiatric problems compared to unguided SSIs [58]. No significant effects were observed for other outcomes in the analyses (all p > 0.05).

We emphasise the small but consistent evidence for gratitude-centered WIs in supporting youth mental health, highlighting their simplicity and ease of implementation. These interventions, which involve writing daily gratitude reflections for brief periods, foster self-efficacy—an important factor for young people [71]. As a practical mental health support option, gratitude interventions are particularly important where access to therapy is limited or stigma limits seeking help, especially considering that youth are among the least likely to pursue professional support [48].

While there are indications that gratitude interventions may be particularly effective, caution is needed. We do not yet know why they are more effective, and their impact can be confounded by other factors. Studies like those by Venturo-Conerly et al. [78] suggest that gratitude may be just as effective as growth mindset interventions, and when tested head-to-head, the results are not always consistent or superior. Based on this, we propose that cultivating gratitude for strengths within stigmatised identities, rather than focusing on negative narratives surrounding these identities, can help alleviate mental health problems and build resilience, as shown in studies with refugee students and individuals with depression [4, 5]. We recommend that intervention developers further explore this promising strategy to enhance mental health among young people with stigmatised identities, particularly in educational settings such as those for international students [46].

Our review also reveals that the gratitude interventions included primarily targeted mental health outcomes rather than educational ones, with a focus mainly on university students. Secondary outcomes assessed aspects of participants’ personal lives, such as life satisfaction and subjective happiness, suggesting that gratitude practices may not only alleviate anxiety and depression but also enhance overall life satisfaction among youth. This improvement could contribute to the documented effectiveness of these interventions in reducing depressive symptoms; however, we were unable to explore potential mediating effects due to the diverse mechanisms identified in the studies.

As researchers investigate the effectiveness of WIs across various outcomes, optimal intervention characteristics for enhancing their impact on young people's mental health will become clearer. Understanding these characteristics is essential for improving WIs and meeting the needs of diverse populations. Our findings highlight the benefits of gratitude-based interventions, especially for those with stigmatised identities who face barriers to accessing professional mental health support [14, 21]. By framing these identities through gratitude and strengths-based approaches, we may alleviate mental health challenges. Overall, WIs demonstrate promise in effectively reaching hard-to-reach populations through their non-stigmatising, engaging, and brief, self-administered design.

### Strengths and Limitations

This review fills a significant gap in the literature by being the first to evaluate the effectiveness of WIs on depressive and anxiety symptoms in young people. It highlights specific intervention moderators and subgroups linked to positive outcomes in youth mental health, marking a new frontier in understanding WIs’ effects. By assessing multiple endpoints, we comprehensively explored the long-term effects of WIs on youth mental health outcomes rather than focusing on a single effect size for specific symptomatology, providing valuable insights for future research and practice.

The findings of this study should be interpreted with caution due to several limitations. As our search was conducted prior to the publication of this study, we recognise that newer research may offer further insights into the effectiveness of WIs for youth depression and anxiety. Future research should aim to incorporate a broader range of recent WI studies to assess the effectiveness of these interventions. Additionally, significant heterogeneity in depressive symptoms was observed at the 3-month follow-up, and the limited sample size restricted our ability to perform a comprehensive moderator analysis while controlling for confounding variables. This prevented us from identifying the most effective combinations of core ingredients, settings, durations, and delivery methods for reducing depressive and anxiety symptoms within a unified model. Moreover, the emerging nature of the literature on these interventions made it impractical to perform a robust statistical analysis of all relevant mediating mechanisms.

The broad age range in this meta-analysis, spanning multiple developmental stages, may also limit the generalisability of our findings. Future research should address this by focusing on narrower age brackets to explore these developmental nuances more effectively. Another limitation is that many of the studies included non-clinical populations (e.g., students), and improvements among individuals with higher baseline scores may reflect regression to the mean rather than the intervention alone. Since our analysis did not control for this phenomenon, its potential influence should be considered when interpreting the results. Moreover, although we used Cohen’s conventional effect size thresholds, widely applied in mental health reviews [10], these benchmarks may be more conservative. In the context of educational interventions, smaller effect sizes are often considered meaningful [3, 33, 88].

Moreover, the reliance on a predominantly WEIRD (Western, Educated, Industrialised, Rich, and Democratic) sample and exclusively English-language studies may introduce bias [28], underscoring the need for future research to include more diverse participants, especially since WIs aim to address inequalities faced by marginalised groups [61]. Additionally, publication bias presents a significant concern that could impact meta-analytic conclusions, as we observed asymmetry across the follow-up points examined in this study [10]. We acknowledge the limitations of our small sample size and reduced statistical power, which restricted a more comprehensive meta-analysis. We encourage readers to critically evaluate the results and conclusions, considering the complexities of systematic reviews and meta-analyses, including the quality and availability of data from primary studies and the inherent limitations of meta-analytic methodologies.

## Conclusion

Our study shows that WIs lead to overall improvements in depressive and anxiety symptoms post-intervention, with sustained reductions in anxiety symptoms observed at 3-month follow-ups. However, we found no consistent effects on either symptom cluster at longer-term follow-ups. Moderator analyses indicated that gratitude-based interventions and online delivery significantly enhanced the effectiveness of WIs, particularly in reducing depressive symptoms. However, due to the limited sample size, these findings should be interpreted with caution. As the popularity of WIs increases, future research should explore which intervention features promote mental health benefits, contributing to the development of more sustainable strategies for supporting youth mental health.

## Supplementary Information

Below is the link to the electronic supplementary material.

Supplementary file1 (PDF 411 KB)

Supplementary file2 (DOCX 12 KB)

Supplementary file3 (XLSX 20 KB)

Supplementary file4 (DOCX 18 KB)

Supplementary file5 (DOCX 27 KB)

Supplementary file6 (DOCX 31 KB)

## Data Availability

The raw data supporting the conclusions of this article will be made available by the authors upon reasonable request.

## Footnotes

1 By active ingredients, we follow Wolpert et al. [87], defining them as “aspects of an intervention that drive clinical effect, are conceptually well defined, and link to specific hypothesised mechanisms of action. In the context of WIs, it refers to a specific psychological process the intervention aims to change—one shift in interpretation— rather than for example, a mode of delivery (e.g., writing exercises) or content domain (e.g., a particular value in a self-affirmation intervention). Unlike multi-component interventions targeting various cognitive, behavioural, or emotional processes, WIs focus on altering one key way people interpret themselves or their experiences. In essence, WIs are designed to address a specific, hypothesised psychological barrier. For example, a social-belonging intervention targets the belief “I don’t belong here,” while a growth mindset intervention challenges the belief that “intelligence is fixed.” Both aim to reframe critical beliefs that influence outcomes like academic performance or well-being. Similarly, although a self-affirmation exercise might focus on different value domains (e.g., family, community), the underlying active ingredient remains the same: affirming an individual’s broader sense of self-integrity, reinforcing the idea that people are not defined by a single threat or stereotype, but by a range of self-defining strengths.

2 Due to lack of relevant data in the included studies, we have not reported on mediation effects although this area of inquiry was initially proposed in the pre-registered review protocol.
