# Supplementary material for: Effectiveness and Moderators of Wise Interventions in Reducing Depressive and Anxiety Symptoms Among Youth: A Systematic Review and Meta-analysis of Randomised Controlled Trials
Source: Child Psychiatry Hum Dev. 2025 Apr 7;57(6):1873–85. doi: 10.1007/s10578-025-01832-4 (PMC13748166; doi:10.1007/s10578-025-01832-4)
Supplement: Supplementary file 2 [file 10578_2025_1832_MOESM2_ESM.docx]

**Online Resource 2**

**Search terms**

summary((adolescen* OR youth* OR young OR teen* OR child* OR pupil* OR student* OR undergrad*))

AND

(("expectancy value*" OR expectancy-value* OR "utility value*" OR utility-value* OR "stress mindset*" OR "stress reappraisal*" OR malleability OR values-alignment* OR "empathetic mindset*" OR "emotional reappraisal*" OR "growth mindset*" OR mindset OR mind-set OR gratitude OR "implicit theor*" OR "lay theor*" OR "incremental theor* " OR "self affirm*" OR self-affirm* OR "self theor*" OR self-theor* OR value-affirm* OR "value affirm*" OR social-belonging OR "social belonging" OR "cross-group friendship" OR "social psycholog*" OR social-psychology* OR "mental contrasting" OR brief OR brief OR single-session OR "self-regulatory") NEAR/3 (intervention* OR program* OR workshop* OR treat* OR prevent* OR teaching OR changing OR training OR manipulation* OR "implementation intention*" OR meeting* OR strategy))

AND

(summary((depressi* OR anxi* OR "social anxi*")) OR summary((mental OR psychologic* OR emotion* OR stress) NEAR/3 (symptom* OR disorder* OR problem* OR outcome* OR stress OR distress OR difficult*)))

AND

(summary("randomi?ed control* trial") OR title("randomi?ed trial") OR summary(randomi?ed) OR summary(randomly)) AND la.exact("ENG") AND la.exact("ENG") AND la.exact("ENG")
