# Supplementary material for: Effectiveness and Moderators of Wise Interventions in Reducing Depressive and Anxiety Symptoms Among Youth: A Systematic Review and Meta-analysis of Randomised Controlled Trials
Source: Child Psychiatry Hum Dev. 2025 Apr 7;57(6):1873–85. doi: 10.1007/s10578-025-01832-4 (PMC13748166; doi:10.1007/s10578-025-01832-4)
Supplement: Supplementary file 3 [file 10578_2025_1832_MOESM4_ESM.docx]

**Online Resource 4**

**Funnel Plots**

**Figure 1**

*The Funnel Plot for Post-Intervention Depressive Symptoms*

**Figure 2**

*The Funnel Plot for 3-Months Depressive Symptoms*
