# Supplementary material for: Effectiveness and Moderators of Wise Interventions in Reducing Depressive and Anxiety Symptoms Among Youth: A Systematic Review and Meta-analysis of Randomised Controlled Trials
Source: Child Psychiatry Hum Dev. 2025 Apr 7;57(6):1873–85. doi: 10.1007/s10578-025-01832-4 (PMC13748166; doi:10.1007/s10578-025-01832-4)
Supplement: Supplementary file 4 [file 10578_2025_1832_MOESM5_ESM.docx]

**Online Resource 5**

**Forest Plots**

**Figure 1**

*Forest Plot of Post-Intervention Depressive Symptoms*

**Figure 2**

*Forest Plot of Post-Intervention Anxiety Symptoms*

**Figure 3**

*Forest plot of 3-months anxiety symptoms*
