# Supplementary material for: Effectiveness and Moderators of Wise Interventions in Reducing Depressive and Anxiety Symptoms Among Youth: A Systematic Review and Meta-analysis of Randomised Controlled Trials
Source: Child Psychiatry Hum Dev. 2025 Apr 7;57(6):1873–85. doi: 10.1007/s10578-025-01832-4 (PMC13748166; doi:10.1007/s10578-025-01832-4)
Supplement: Supplementary file 5 [file 10578_2025_1832_MOESM6_ESM.docx]

**Online Resource 6**

**Subgroup Analyses Tables across Timepoints**

| **Table 1**  *Subgroup analyses for the post-intervention time point* | | | | | | | | | | | |
| --- | --- | --- | --- | --- | --- | --- | --- | --- | --- | --- | --- |
| WI characteristics | | Depressive symptoms | | | | | Anxiety symptoms | | | | |
|  |  | K | Point estimate (Hedges’ g) | P-value | 95% CI Lower Limit | 95% CI Upper Limit | K | Point estimate (Hedges’ g) | P-value | 95% CI Lower Limit | 95% CI Upper Limit |
| WI type | Overall | 8 | 0.22 | 0.001 | 0.09 | 0.34 | 6 | 0.20 | <0.001 | 0.06 | 0.33 |
|  | Gratitude | 4 | 0.38 | 0.001 | 0.16 | 0.60 | 3 | 0.30 | 0.02 | 0.05 | 0.54 |
|  | Self-affirmation | 3 | 0.09 | 0.28 | -0.07 | 0.26 | 2 | 0.18 | 0.04 | 0.01 | 0.36 |
|  | Social belonging | 1 | 0.38 | 0.08 | -0.04 | 0.81 | - | | | | |
|  | Growth mindset | - | | | | | 1 | 0.06 | 0.72 | -0.27 | 0.39 |
| Setting | Overall | 8 | 0.22 | 0.001 | 0.09 | 0.34 | 6 | 0.20 | <0.001 | 0.06 | 0.33 |
|  | Home | 2 | 0.26 | 0.09 | -0.04 | 0.56 | 2 | 0.26 | 0.08 | -0.03 | 0.56 |
|  | Laboratory | 2 | 0.20 | 0.23 | -0.13 | 0.53 | 1 | 0.06 | 0.72 | -0.27 | 0.39 |
|  | Online | 3 | 0.43 | 0.001 | 0.18 | 0.69 | 2 | 0.21 | 0.19 | -0.10 | 0.53 |
|  | School | 1 | 0.08 | 0.38 | -0.10 | 0.27 | 1 | 0.21 | 0.03 | 0.02 | 0.40 |
| Method of delivery | Overall | 8 | 0.22 | 0.001 | 0.09 | 0.34 | 6 | 0.20 | <0.001 | 0.06 | 0.33 |
|  | Self-administered | 7 | 0.32 | <.001 | 0.15 | 0.48 | 5 | 0.19 | 0.04 | 0.01 | 0.37 |
|  | Guided | 1 | 0.08 | 0.38 | -0.10 | 0.27 | 1 | 0.21 | 0.03 | 0.02 | 0.40 |
| Duration | Overall | 8 | 0.22 | 0.001 | 0.09 | 0.34 | 6 | 0.20 | <0.001 | 0.06 | 0.33 |
|  | Multiple | 5 | 0.36 | <.001 | 0.16 | 0.55 | 4 | 0.24 | 0.03 | 0.02 | 0.46 |
|  | Single | 3 | 0.11 | 0.18 | -0.05 | 0.28 | 2 | 0.17 | 0.04 | 0.01 | 0.34 |

**Table 2**

*Subgroup analyses for the 3-month follow-up time point*

| WI characteristics | | Depressive symptoms | | | | | Anxiety symptoms | | | | |
| --- | --- | --- | --- | --- | --- | --- | --- | --- | --- | --- | --- |
|  |  | K | Point estimate (Hedges’ g) | P- value | 95% CI Lower Limit | 95% CI Upper Limit | K | Point estimate (Hedges’ g) | P- value | 95% CI Lower Limit | 95% CI Upper Limit |
| WI type | Overall | 8 | 0.08 | 0.40 | -0.11 | 0.28 | 6 | 0.09 | 0.02 | 0.01 | 0.18 |
|  | Gratitude | 2 | 0.05 | 0.81 | -0.37 | 0.48 | 1 | 0.20 | 0.36 | -0.24 | 0.65 |
|  | Growth mindset | 4 | 0.03 | 0.83 | -0.22 | 0.27 | 4 | 0.09 | 0.04 | 0.01 | 0.17 |
|  | Self-affirmation | 1 | 0.20 | 0.53 | -0.42 | 0.82 | 1 | 0.10 | 0.66 | -0.36 | 0.56 |
|  | Social belonging | 1 | 0.35 | 0.25 | -0.25 | 0.94 | - | | | | |
| Setting | Overall | 8 | 0.08 | 0.46 | -0.14 | 0.30 | 6 | 0.09 | 0.02 | 0.01 | 0.18 |
|  | Home | 2 | 0.05 | 0.83 | -0.42 | 0.53 | 1 | 0.20 | 0.36 | -0.24 | 0.65 |
|  | Laboratory | 2 | 0.21 | 0.36 | -0.25 | 0.68 | 1 | 0.24 | 0.24 | -0.16 | 0.64 |
|  | Online | 3 | 0.01 | 0.96 | -0.33 | 0.34 | 3 | 0.08 | 0.08 | -0.01 | 0.17 |
|  | School | 1 | 0.14 | 0.63 | -0.43 | 0.72 | 1 | 0.12 | 0.37 | -0.15 | 0.39 |
| Method of delivery | Overall | 8 | 0.08 | 0.34 | -0.08 | 0.24 | 6 | 0.09 | 0.02 | 0.01 | 0.18 |
|  | Self-administered | 8 | 0.08 | 0.34 | -0.08 | 0.24 | 6 | 0.09 | 0.02 | 0.01 | 0.18 |
| Duration | Overall | 8 | 0.08 | 0.37 | -0.10 | 0.26 | 6 | 0.09 | 0.02 | 0.01 | 0.18 |
|  | Multiple | 3 | 0.10 | 0.56 | -0.23 | 0.43 | 2 | 0.16 | 0.34 | -0.16 | 0.47 |
|  | Single | 5 | 0.07 | 0.49 | -0.13 | 0.28 | 4 | 0.09 | 0.04 | 0.01 | 0.17 |

**Table 3**

*Subgroup analyses for the 6-month follow-up time point*

| WI characteristics | | Depressive symptoms | | | | | Anxiety symptoms | | | | | | | | | | |
| --- | --- | --- | --- | --- | --- | --- | --- | --- | --- | --- | --- | --- | --- | --- | --- | --- | --- |
|  |  | K | Point estimate (Hedges’ g) | P-value | 95% CI Lower Limit | 95% CI Upper Limit | K | Point estimate (Hedges’ g) | | P-value | | | 95% CI Lower Limit | | 95% CI Upper Limit | | |
| WI type | Overall | 6 | 0.10 | 0.07 | -0.01 | 0.21 | 2 | 0.07 | | 0.52 | | | -0.14 | | 0.27 | | |
|  | Growth mindset | 5 | 0.09 | 0.12 | -0.02 | 0.20 | 2 | 0.07 | | 0.52 | | -0.14 | | | | 0.27 | |
|  | Social belonging | 1 | 0.25 | 0.24 | -0.17 | 0.68 | - | | | | | | | | | | |
| Setting | Overall | 6 | 0.10 | 0.07 | -0.01 | 0.21 | 2 | 0.07 | | 0.52 | | -0.14 | | | | 0.27 | |
|  | Laboratory | 2 | 0.30 | 0.06 | -0.01 | 0.06 | 1 | 0.20 | | 0.32 | | -0.20 | | | | 0.60 | |
|  | Online | 1 | -0.10 | 0.41 | -0.33 | 0.14 | 1 | 0.02 | | 0.87 | | -0.22 | | | | 0.25 | |
|  | School | 3 | 0.12 | 0.08 | -0.01 | 0.25 | - | | | | | | | | | | |
| Method of delivery | Overall | 6 | 0.10 | 0.07 | -0.01 | 0.21 | 2 | | 0.07 | | 0.52 | | | -0.14 | | | 0.27 |
|  | Self-administered | 3 | 0.06 | 0.54 | -0.13 | 0.24 | 2 | | 0.07 | | 0.52 | | | -0.14 | | | 0.27 |
|  | Guided | 3 | 0.12 | 0.08 | -0.01 | 0.25 | - | | | | | | | | | | |
| Duration | Overall | 6 | 0.10 | 0.07 | -0.01 | 0.21 | 2 | | 0.07 | | 0.52 | | | -0.14 | | | 0.27 |
|  | Multiple | 3 | 0.12 | 0.08 | -0.01 | 0.25 | - | | | | | | | | | | |
|  | Single | 3 | 0.06 | 0.54 | -0.13 | 0.24 | 2 | | 0.07 | | 0.52 | | | -0.14 | | | 0.27 |
